# Natural toxins and One Health: a review

**DOI:** 10.1016/j.soh.2023.100013

**Published:** 2023-03-07

**Authors:** Azubuike Raphael Nwaji, Onikisateinba Arieri, Annabel Sharon Anyang, Kaze Nguedia, Etomi Barbara Abiade, Gilead Ebiegberi Forcados, Olusola Olalekan Oladipo, Sunday Makama, Ishaku Leo Elisha, Nonyelim Ozele, Jurbe Gofwan Gotep

**Affiliations:** a Department of Physiology, Faculty of Basic Medical Sciences, Alex Ekwueme Federal University, Ndufu-Alike, Ebonyi State, Nigeria; b Department of Industrial Chemistry and Petrochemical Technology, Faculty of Science Laboratory, University of Portharcourt, Nigeria; c Faculty of Veterinary Medicine, University of Abuja, Nigeria; d Department of Biochemistry, Faculty of Medicine and Biomedical Sciences, University of Yaounde I, Cameroon; e Faculty of Pharmaceutical Science, University of Jos, Plateau State, Nigeria; f Biochemistry Division, National Veterinary Research Institute, Vom, Plateau State, Nigeria; g Drug Development Division, National Veterinary Research Institute, Vom, Plateau State, Nigeria

**Keywords:** One Health, Mycotoxins, Phytotoxins, Phycotoxins, Insect toxins, Aqua toxins

## Abstract

**Background:**

The One Health concept considers the interconnectivity, interactions and interdependence of humans, animals and the environment. Humans, animals and other organisms are constantly exposed to a wide range of natural toxins present in the environment. Thus, there is growing concern about the potential detrimental effects that natural toxins could pose to achieve One Health. Interestingly, alkaloids, steroids and bioactive peptides obtained from natural toxins could be used for the development of therapeutic agents.

**Methodology:**

Our literature search focused on the following keywords; toxins, One Health, microbial toxins, mycotoxins, phytotoxins, phycotoxins, insect toxins and toxin effects. Google Scholar, Science Direct, PubMed and Web of Science were the search engines used to obtain primary databases. We chose relevant full-text articles and review papers published in English language only. The research was done between July 2022 and January 2023.

**Results:**

Natural toxins are poisonous substances comprising bioactive compounds produced by microorganisms, invertebrates, plants and animals. These compounds possess diverse structures and differ in biological function and toxicity, posing risks to human and animal health through the contamination of the environment, causing disease or death in certain cases. Findings from the articles reviewed revealed that effects of natural toxins on animals and humans gained more attention than the impact of natural toxins on the environment and lower organisms, irrespective of the significant roles that lower organisms play to maintain ecosystem balance. Also, systematic approaches for toxin control in the environment and utilization for beneficial purposes are inadequate in many regions. Remarkably, bioactive compounds present in natural toxins have potential for the development of therapeutic agents. These findings suggest that global, comprehensive and coordinated efforts are required for improved management of natural toxins through an interdisciplinary, One Health approach.

**Conclusion:**

Adopting a One Health approach is critical to addressing the effects of natural toxins on the health of humans, animals and the environment.

## 1 Introduction

The complex relationship between humans, animals and the environment has resulted in a human–animal–environment interface that is so intrinsically linked [1]. The One Health concept considers the interactions between humans, animals and the environment, recognizing the fact that human health is closely linked to animal and environmental health [2]. It demonstrates the inseparability and interdependence of human, animal and environmental health through a unified view of health care management systems [3]. The One Health concept also formulates clearly both the need for and the benefit of cross-sectoral collaborations [4]. It also deals with the challenges at the intersection of animal, human and environmental health [2].

Natural toxins are compounds that are naturally produced by living organisms from different kingdoms of life, possessing a wide range of biological functions and activities that could be detrimental to human and animal health [5]. These bioactive compounds are generally produced by the respective organisms to confer competitive advantages in the environment in which they live and enhance survival [6]. The mechanism of action of these natural toxins range from proteolytic, coagulant, hemolytic or neurotoxic activities [7]. Interestingly, natural toxins could be beneficial to human and animal health as some xenobiotics obtained from these toxins are currently used for therapeutic purposes [8]. Natural toxins could be classified based on biological origin, target organ toxicity or mode of action [9].

Natural toxins are generally produced to ward off predators, protect colonies and capture prey [10]. These toxins range from cardioactive steroids [11], cardiac glycosides [12] and bioactive peptides [13]. The mechanism of action of natural toxins include inhibition of sodium-potassium ATPase, inhibition of angiotensin converting enzyme , binding to ion channels, binding to and inactivating proteins [14]. Toxins also differ in terms of resistance to heat and sun, as some are heat labile while others are not, thereby affecting their bio-accumulation potential [15]. Some toxins are harmful to certain species while other species are unharmed by them, like the spider venom which is poisonous to many animals but not poisonous to lizards [16]. The major symptoms associated with natural toxin poisoning include nausea, pain, gastrointestinal disorders, cardiotoxicity, paralysis, hepatotoxicity and neurotoxicity [[17], [18], [19]]. Studies have also examined benefits that could be obtained from natural toxins with respect to developing antimicrobial and anticancer agents [8,20].

Zootoxins are a class of toxins produced by venomous animals which can cause toxicity in plants, animals and humans [7]. Snake envenomation is widely reported among terrestrial animals [21]. It is reported that snake envenomation affects millions of people resulting in about 100,000 deaths annually [22]. Morbidities caused by snake envenomation is attributed to metalloproteases contained in the venom that degrade cells and tissues resulting in loss of limbs or paralysis [22]. Cytotoxins present in cobra venom cause cell apoptosis and necrosis by altering membrane permeability as well as induce heart failure by depolarizing neurons and cardiocyte membranes [23]. A retrospective study on hospital admissions in Cyprus due to snake envenomation reported a number of morbidities and two mortality cases [24]. A retrospective study on snake envenomation in dogs reported collapse, paresis and death as effects of snake envenomation in dogs [25]. A similar study in Korea reported echinocytosis, anemia and acute kidney injury in dogs that suffered from snake envenomation [26]. Snake envenomation in cats has also been reported [27] with coagulopathy and hemorrhage observed [28]. It is noteworthy that snake venoms could be beneficial, as components in snake venoms are used for the management of diseases [20]. Specifically, studies have reported that components present in snake venom possess antimicrobial and antitumor properties [21].

This review focuses on natural toxins produced by plants, bacteria, fungi, insects and aquatic organisms. The fact that humans and animals are exposed to a variety of natural toxins in their environment [29] with potential adverse effects [30] is evidenced from recent public health emergencies caused by zoonotic diseases [31]. Currently, the major limitations associated with natural toxin management include inadequate analytical standards, testing methods and comprehensive databases [12]. Therefore, this review provides an insight into the diversity of natural toxins, emphasizing how these toxins impact the health of humans, animals and the environment. We also identify gaps that need to be addressed in order to ensure sustainable global health.

## 2 Phytotoxins and One Health

Phytotoxins are poisonous substances synthesized by plants through naturally occurring biochemical reactions [32]. They are secondary metabolites produced by plants which act as defense tools [33]. They are phytochemicals from plants that act as xenobiotic to the environment, animals and humans, occasionally causing adverse effects to humans, animals and the environment [34]. Globally, the use of medicinal plants for the management of human and animal health is gaining more interest due to concerns of drug resistance caused by misuse and abuse of conventional drugs in animal and human health medicine [35]. Notwithstanding these promising therapeutic benefits, there are still adverse side effects in the form of phytotoxins that could be detrimental to human and animal health. Such phytotoxins include lectins, glycosides, cyanogen, safrole, solanine, ricininechaconine and other alkaloids [36]. These phytotoxins vary in composition and mode of action [37].

### 2.1 Effects of phytotoxins in humans and animals

Lectins are multivalent carbohydrate-binding proteins found mostly in legumes which cause agglutination of particular cells or precipitation of glycoconjugates and polysaccharides [38]. Lectin consumption in humans is associated with symptoms like nausea, abdominal cramp, pain and swollen joints [39]. In animals, high dietary lectin intake can impair nutrient utilization and also cause damage to gut epithelium leading to digestive disorders [40]. Despite the toxic nature of lectins to animals and humans, they have reportedly shown some health advantages with the intervention of biotechnology as they are used to detect specific red cell antigens, activate lymphocytes and detect surface markers of stem cells [38]. Lectins have also shown antimicrobial effects and could be used for the development of efficacious antimicrobial agents [41].

Alkaloids are natural occurring organic compounds mostly found in roots, fruits and stems that contain organic nitrogen atoms [42]. Plants secrete alkaloids during secondary metabolism as defense mechanism against predators which could become toxic to humans and animals at high concentration and frequent exposure [43]. Pyrrolizidine alkaloids (PA) are reported to have adverse effects on humans and animals by inducing liver damage [44]. Excess consumption of green tea rich in pyrrolizidine that could cause adverse effects to the liver due to metabolic transformation processes in the liver [45]. However, alkaloids could be useful for drug development as the presence of nitrogen in the alkaloid structure enhances their drug properties [46]. For example, quinine and artemisinin are useful for the management of viral diseases and other parasitic infections [47].

Glycosides are found in flowers and fruit pigments with a carbohydrate portion consisting of one or more sugars (glycone) or a non-sugar (aglycone) moiety combined with hydroxyl compound [48]. However, cardiac glycosides are toxic to animals and humans [49]. Glycoalkaloid toxicity in potatoes has also been reported [50]. The hydrolysis of cyanogenic glycosides during food processing or crushing forms nitrogen cyanide which is toxic to humans and animals [51]. A study examined the presence of hydrogen cyanide in the form of cyanogenic glycoside in many plant samples and reported the presence of cyanide in many plants [52]. Cyanide prevents cells from utilizing oxygen, thereby leading to apoptosis of cells mostly of the heart and brain [53]. Interestingly, glycosides could serve medicinal purposes as antibiotics (streptomycin) and also be used for treatment of heart related diseases [48].

Ricinine, found mostly in castor beans, is a carbohydrate-binding protein associated with adverse health effects in humans [54]. It impairs optimal utilization of proteins during metabolism [55]. A study examined the effect of castor beans oil in a dietary feed formulation on chicks and observed that the final weight of the chicks reduced against the control, while the white blood cell and platelet counts were also affected suggesting the adverse effects of ricinine in castor bean [56]. The beneficial potential of ricinine could be in its insecticidal properties, where insect populations constitute pests of significant economic value [57].

Safrole is a slightly yellowish oily liquid mostly found from root, bark and fruit of certain plants like *Ocoteaodorifera* [58]. Safrole is carcinogenic and can also cause liver damage if consumed in large quantity [59]. Eisenreich et al. [58] also reported the toxic and carcinogenic properties of safrole with symptoms of safrole toxicity including vomiting, stupor, vertigo and pallor if consumed excessively. Interestingly, a recent study reported that safrole could be used as an antioxidant and for the management of diabetes [60].

Phytoestrogens are plant-derived compounds that could affect fertility output in animals and cause cancer in humans [61]. They alter physiological signaling pathways by interacting with estrogen receptors α, β and G-protein coupled estrogen receptors, thereby altering downstream functions implicated in disease pathogenesis [62]. The effects of dietary phytoestrogens on mouse testis was evaluated in a study and results suggested that phytoestrogens might have detrimental effects on male reproductive function in a dose-dependent manner [63]. The benefits of phytoestrogens include correcting hormonal imbalance and managing endocrine disruption in postmenopausal women who experience lower physiological estrogen levels [64].

### 2.2 Effect of phytotoxins on the environment

Phytotoxins affect the environment by introducing toxic compounds into the environment, contributing to complex mixture of organic micro pollutants [5]. This in turn inhibits the growth of other economical organisms and plants in the ecosystem. They also contribute to soil-borne diseases that inhibit the growth of commercial crops [65]. Phytotoxins can introduce inherent toxins to soil when leaves, stems and branches fall to the ground [66]. Water currents during rainfall can subsequently wash these plant tissues to water bodies, thereby introducing these phytotoxins to aquatic animals with tendency for bioaccumulation [66].

Rotenone, a phytotoxin found in sunflower seeds as well as plants of the *Derris* and *Tephrosia* species, is a known environmental pollutant which causes damage to silk worm insects, snails, fish and mice [67]. A study carried out in mouse neural stem cells showed that exposure to rotenone caused altered mitochondrial function and induced ferroptosis in neurons and associated alterations to normal physiological functions [67]. A short-term toxicity study carried out on fish showed that exposure to rotenone caused a wide range of toxicities, necessitating further chronic exposure studies [68]. Another study on selected invertebrate groups reported that exposure to rotenone caused significant toxicity to planktonic invertebrates which could be exposed to this phytotoxin in nature during biocontrol activities that utilize rotenone [69].

**Fig. 1:**
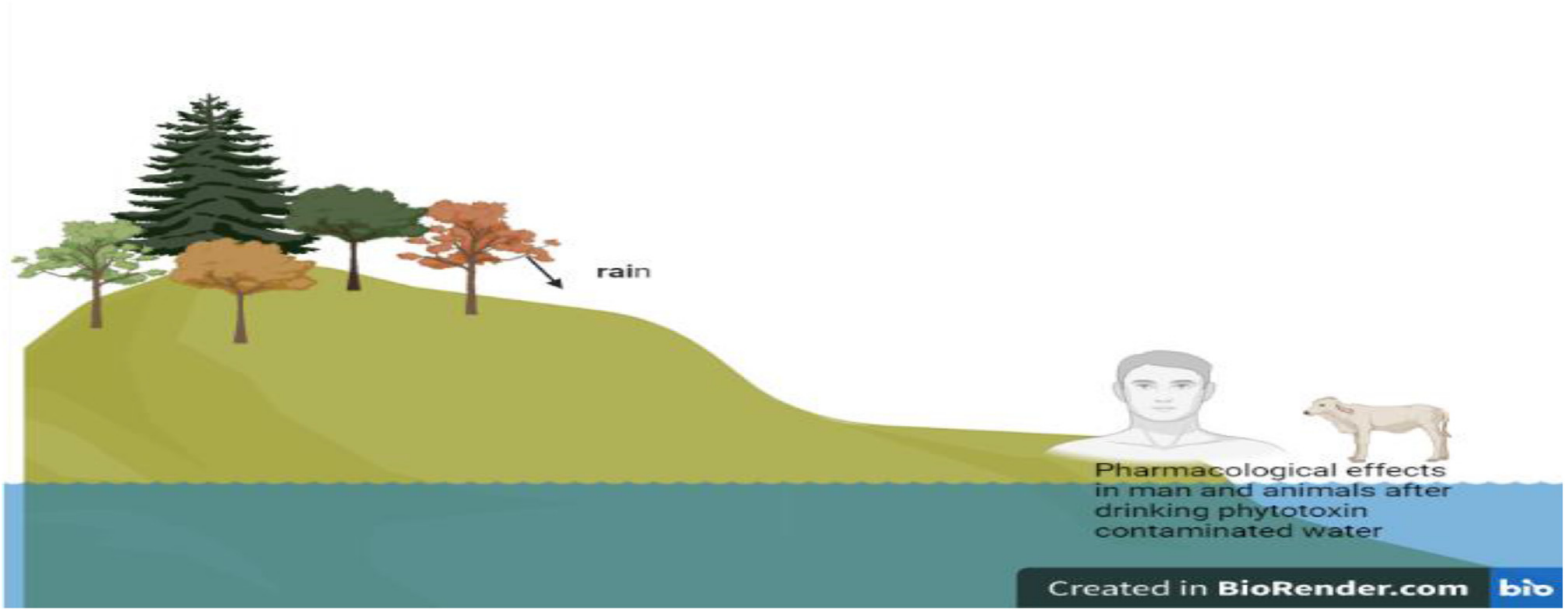
Phytotoxins in humans, animals and the environment.

Azadirachtin is a phytotoxin present in *Azadirachta indica* (Neem tree), which has been shown to be toxic to insects that feed on the leaves, soil flora like nematodes when the leaves and stems fall to the ground and aquatic organisms including fishes when the leaves and stems are washed into water bodies [70]. Seeds from leguminous plants which are rich in sulfur could be washed into water bodies, undergo decomposition to cause elevated sulfur concentrations in water bodies and cause toxicity to aquatic organisms [71]. Sulfide is regarded as a soil phytotoxin with a wide range of effects on various ecosystems [72]. A risk assessment study showed that 34% of 1586 phytotoxins present in 844 plant species showed aquatic pollutant potential, stressing the need for further environmental pollutant assessment. These reports point to the fact that phytotoxins with a potential to bioaccumulation could pass across ecosystems and food chains, posing risks as environmental pollutants as illustrated in Fig. 1 [73]. Further studies are needed in this regard that especially focus on the effects of phytotoxins on soil and water microbes as well as ecosystem balance.

## 3 Microbial toxins and One Health

Microorganisms are ubiquitous, producing a range of bioactive compounds such as antibiotics, hydrolytic enzymes and toxins [6]. Microbial toxins are implicated in infection and disease through direct damage to host tissues and impairing the immune system. Based on target biological effects, microbial toxins can be classified as enterotoxins, cytotoxins, neurotoxins, leukotoxins, dermonecrotic toxins, and hemolytic toxins [74]. The sources of microbial toxins could be from prokaryotes such as bacteria and eukaryotes such as diatoms, dinoflagellates and fungi [6]. These microbial pathogens disrupt host signaling pathways and host cell structures to establish and maintain infection and achieve these through numerous toxin-dependent mechanisms [75]. Microbial toxins are of significant importance to humans, animals, plants and the environment [76]. There are two types of toxins produced by bacteria, the cell-associated lipopolysaccharide toxins released following cell destruction are known as endotoxins. In contrast, the toxic proteins synthesized inside the cell and released to target cells are known as exotoxins [77]. A specific bacterium may produce a single toxin or multiple toxins, such as in *Bacillus cereus*, *Clostridium botulinum*, *Clostridium perfringens* and *Staphylococcus aureus* [78]. Under the umbrella of bacterial exotoxins, there are pore-forming toxins and heat-stable enterotoxins. Toxins in the former category include listeriolysin O (*Listeria monocytogenes*), hemolysins (*Escherichia coli*), alpha-toxin (*Staphylococcus* spp.), and cholesterol-dependent cytolysins. In contrast, the latter category includes toxins produced by microbes such as *Klebsiella* and *Vibrio* [79].

Clostridium species are a diverse group of Gram-positive, anaerobic spore-forming bacteria widely spread in nature, producing more toxins than any other known bacteria [77]. Botulinum neurotoxins (BoNTs) produced by *C. botulinum* as well as atypical strains of *Clostridium* spp., such as *C. butyricum* and *C. baratii* are poisonous [80]. The BoNTs are a family of bacterial protein toxins that cause a devastating disease in humans and animals known as botulism [76]. Botulism is a global public health concern due to its extreme lethal capability and potential as a biological weapon [80]. Another member of this genus is the bacterium *Clostridium tetani* that grows anaerobically at the site of an injury, in the process producing the extremely potent tetanus neurotoxin (TeNT), which induces tonic muscle spasm, paralysis and eventual death through the blockade of inhibitory neurotransmitters in the spinal cord. In addition, *C. perfringens* produces several toxins implicated in several histotoxic and enterotoxic effects [81]. *C*. *perfringens* is categorized into type A, B, C, D and E. Type A which causes gastroenteritis is the most potent [82].

*Staphylococcal species* are Gram-positive facultative anaerobic bacteria commonly found in food due to environmental, human and animal contamination [77]. This pathogen manipulates the innate and adaptive immunity of the host due to a plethora of virulence factors, many of which are highly inflammatory secreted toxins [83]. Staphylococcal enterotoxins are one of the most notable virulence factors associated with *S. aureus*; they are potent gastrointestinal toxins implicated in emesis and interestingly termed super antigens due to their stimulation of non-specific T-cell proliferation [78]. There are over 20 distinct single-chain exotoxins chromosomally encoded by *S*. *aureus*. They are primarily implicated in food poisoning either due to food contamination with preformed exotoxins or with exotoxin-producing strains [84].

Shiga toxin (Stx), considered one of the most potent biological poisons known, is produced by *Shigella dysenteriae* and by some serogroups of *E. coli* (termed Stx1 in *E.coli*) [85]. These pathogens produce toxins that exert their toxigenic activity by protein synthesis inhibition [79]. *B. cereus* is a Gram-positive, facultative aerobic, spore-forming rod shaped bacterium that produces two toxins: the heat-labile toxin similar in effect to *C. perfringens* and the heat-stable toxin responsible for severe emetic intoxication [82]. *Vibrio* species are aquatic toxin producers commonly implicated in seafood-borne infections due to their production of enterotoxins and hemolysins. Other pathogens such as *Campylobacter* spp., *Legionella pneumophila* and *Aeromonashydrophilia* are also important aquatic toxin producers and contaminants of water [6].

### 3.1 Effect of microbial toxins on humans and animals

The ubiquitous nature of microorganisms makes them central to food safety and public health issues due to their link to water and food-borne intoxications [6]. Furthermore, bacteria and their toxins are hardy and cannot be destroyed entirely due to their vast survival and adaptation potential [84]. Toxins produced by bacteria (such as *E. coli* and *S. aureus)* have severe, widespread and often life-threatening impacts on human and animal health. Manifestations of *E. coli* toxico-infection include a range of illnesses such as severe diarrhea, colic, hemorrhagic colitis, thrombotic thrombocytopenia and the leading cause of acute renal failure in children, hemolytic uremic syndrome.

It is suggested that that staphylococcal exfoliative toxins are the etiology behind staphylococcal scalded skin syndrome, including Ritter's disease, toxic epidermal necrosis, bullous impetigo and some incidences of erythema. Predominantly susceptible groups include neonates, infants and immunocompromised adults, while rapid fatality upon intravenous toxin administration is reported in animals. Staphylococcal enterotoxin B is regarded as the most notable etiology for toxic shock syndrome , an often-fatal condition characterized by pyrexia, hypotension and multiorgan system failure [75].

Due to their short incubation periods and massive toxicity, botulinum neurotoxins have potential for use as bio-warfare agents [75]. The botulinum neurotoxins are a family of proteinaceous bacterial toxins [86]. These toxins interfere with neurotransmission of the cholinergic type leading to eventual death by failure of peripheral mechanisms (respiratory failure and cardiac arrest) after a range of manifestations such as nausea, vomiting, diarrhea, muscle fatigue and muscle paralysis [9]. There has also been a report of criminal use of toxins [87]. Some studies have implicated microbial toxins in carcinogenesis, especially in individuals that are in constantly exposed to toxin contaminated food and water [88].

### 3.2 Effect of microbial toxins on the environment

The environment, which is the platform where all forms of life interact, plays a role in toxin transmission [89]. Microbial toxins produced on plant surfaces can be washed by rain into water bodies, resulting in water pollution [90]. Moulds can cause decreased water quality due to the production of off-flavors, discoloration of water and accumulation of surface scums [91]. Microbial toxins can bio-accumulate and get transferred to higher trophic levels of the food chain [92], contributing to microbial toxin propagation especially in invertebrate species [93]. Airborne microbial toxins can reduce air quality and cause diseases.

## 4 Mycotoxins and One Health

Mycotoxins are toxic compounds that are naturally produced by fungi which grow on numerous foodstuffs and feedstuffs such as cereals and legumes [94]. Contamination of food and feed can occur before harvest, during harvest and during storage, especially under warm, damp and humid conditions [95]. The most commonly observed mycotoxins include aflatoxins, ochratoxin A, patulin and fumonisins, which represent a considerable risk to animal and human life [95]. Most mycotoxins are chemically stable and can survive during and after food processing. The most hazardous mycotoxins are produced by fungal species, mainly in the genera *Aspergillus*, *Penicillium* and *Fusarium* [95]. Due to mycotoxin contamination, the global competiveness for agricultural commodities from Sub-Sahara Africa is reduced [95]. Mycotoxin contamination in food and feed negatively impacts health and productivity in humans and animals, causing economic losses for the affected countries [95].

### 4.1 Effects of mycotoxins on humans and animals

Aflatoxins are a class of mycotoxins produced mainly by *Aspergillus flavus and A. parasiticus* [96]. The most potent aflatoxin is aflatoxin B1(AFB1), which causes liver damage through cell cycle arrest, damage to cellular macromolecules, induction of apoptosis, oxidative stress and autophagy [96]. AFB1 cause necrosis and degeneration of hepatic parenchyma in birds, fish, non-human primates and rodents by inhibiting the expression of IL-4 that has anti-inflammatory activity and increases the release of IFN-γ and TNF-α from natural killer cells that accelerates inflammatory process [97]. The symptoms associated with aflatoxicosis include pyrexia, anorexia, malaise, pain, nausea and hepatomegaly [94]. In developing countries, inadequate storage infrastructure and humid environmental conditions contribute to aflatoxin contamination and concomitant effects [97].

Ochratoxins are another class of mycotoxins produced by *Aspergillus and Penicillium* genera [98]. Ochratoxins are nephrotoxic, hepatotoxic and teratogenic and carcinogenic in humans and animals [98]. A study reported that Ochratoxin-A contaminated food is associated with human diseases like chronic interstitial nephropathy, Balkan endemic nephropathy and other renal anomalies [99]. Fumonisin is a mycotoxin produced mainly by *Fusarium verticillioides* and *Fusarium proliferatum* [100]. Fumonisin inhibits sphinganine-N-acetyl transferase, involved in sphingolipid metabolism resulting in increased sphinganine and sphingosine along with a decrease in sphingolipid complex, which is the commonly accepted mechanism for fumonisin toxicity [94]. In humans, consumption of fumonisin contaminated sorghum and maize could lead to abdominal pain, borborygmi, diarrhea, reduction of folic acid uptake and the production of developmental defects of neural tube and esophageal cancer [101]. A study carried out in chickens fed safe doses of fumonisin showed altered sphingolipid profiles with histological changes also observed in the liver [102]. Another study in chickens reported that fumonisin B1 can accumulate in tissues, with implications for human exposure through consumption of chickens fed fumonisin contaminated feed [103]. Patulin is another mycotoxin mainly produced by *Aspergillus* and *Penicillium,* found in apples and apple products, moldy fruits, grains and other foods. The acute symptoms in animals include liver, spleen and kidney damage and toxicity to the immune system. For humans, nausea, gastrointestinal disturbances and vomiting have been reported. Interestingly, patulin has medicinal benefits for use as an antibiotic.

### 4.2 Effect of mycotoxins on the environment

Moulds constitute indoor and outdoor air pollutants, especially during seasons of high moisture. Challenges associated with climate change contribute to fungi growth and associated effects [89]. Studies have reported the adverse effects of fungi-contaminated environment, especially fusarium on the respiratory system. Mycotoxins are partially soluble in water and can cause water contamination which can cause environmental pollution [94].

## 5 Insect toxins and One Health

Insects are a large class of living things which secrete venoms or toxins with a lot of toxicological and pharmacological effects [8]. Toxins secreted by insects range from polyamines and peptides to other small molecules which can alter nervous system and ion channel functions in animals, sometimes resulting in morbidity or mortality [8]. However, some of these secretions are pharmacologically beneficial for the management of different diseases [104,105]. One of the most studied classes of toxin- producing insects belong to the order Hymenoptera which prominently includes bees, wasps and ants [105,106]. The venoms from Hymenopterans contain peptides, amines, alkaloids, salts, formic acid and sugars that affect cellular processes leading to inflammation, pain and sometimes paralysis [105].

Another class of toxin-producing insects are spiders, which are invertebrates belonging to the order Araneae [107,108]. The venoms from spiders are mainly peptides which are able to interact with cellular receptors and alter the activity of ion channels, occasionally resulting in inflammation and pain [107]. Centipedes are also a class of insects which produce toxins for defense and predation. Members of the order Scolopendromorpha and Scutigeromorpha are of medical significance [109]. Centipede venoms basically consist of peptides that act as neurotoxins to modulate ion channel function resulting in swelling, pain, cell necrosis or mortality depending on the exposed specie [110]. Scorpions are well known toxin-producing insects of the order Scorpiones, which produce potent venoms for defense and capturing prey [111]. The neurotoxins produced by scorpions can cause altered ion function and neuronal damage resulting in morbidity and cases of mortality especially in children [112].

Interestingly, studies have also examined the medicinal benefits that could be derived from toxins produced by insects [8]. Api-toxin, which is the bee venom, has antioxidant, anti-inflammatory, antifungal and antimicrobial properties, justifying the utilization for the management of inflammatory diseases and wound healing [113]. Bee venom has also been shown to enhance immune function which is necessary for the management of viral diseases caused by HIV and possibly SARS-CoV-2 [114]. Peptides like mastoparan, isolated from wasp venom reportedly exerts antimicrobial, anti-tumor, anti-inflammatory and anticoagulant effects [[115], [116], [117]]. Non-peptide acyl polyamines obtained from spider venom was shown to exert antimicrobial properties against *E. coli* and *S. aureus* [118]. Proteins isolated from centipede venoms are found to be disulfide-rich, with potential antioxidant, anti-inflammatory and therapeutic benefits [119]. Bioactive peptides isolated from centipede venoms also show antimicrobial, apoptotic, antioxidant, anti-inflammatory and anticancer properties [109]. Bioactive compounds isolated from scorpion venom have been used as anticancer agents undergoing phase I and phase II clinical trials, pointing to the medicinal benefits of compounds present in scorpion venom [112].

### 5.1 Effect of insect toxins on humans and animals

Bees are an aggressive class of insects which are known to sting multiple times, releasing venoms that could cause necrosis of skin cells, injury to muscle cells (rhabdomyolysis) and kidney failure that sometimes results in death of patients [120,121]. The main lethal component of bee venom is melitin in addition to other components like phospholipase A2, apamin, histamine, dopamine and hyaluronidase which can cause damage to red blood cells and renal cells leading to ischemic renal lesion and acute tubular necrosis [122]. Clinicians in Brazil reported the case of a three-year old boy who suffered multiple stings from a large swarm of bees and presented with ocular, hepatic, cardiac and renal dysfunction showing that bee stings can result in multiple organ dysfunction [120]. Clinicians in the USA reported the case of a ninety-year old man who suffered multiple stings from bees and presented with facial angioedema, acute kidney injury and rhabdomyolysis [121]. However, pharmacological research is examining how some compounds found in bee toxins could be used to generate medicinal agents of benefit to human health [115].

Wasps also possess an aggressive nature and can sting multiple times without losing their stinger in the process unlike bees [123]. Wasp envenomation can be very painful resulting in multiple tissue dysfunctions as shown from the case report of a forty eight-year old man from Sri Lanka who suffered from multiple wasps stings and presented with acute fulminant hepatitis, acute kidney injury, muscle injury and low platelet count [124]. Also in Sri Lanka, a pregnant thirty nine-year old lady who suffered from multiple wasp sting showed facial edema, myoglobinuria, hemoglobinuria, acute kidney injury and Kounis syndrome [125]. Acute kidney injury is widely reported in patients who suffer from multiple wasp stings [123]. Acute kidney injury attributed to acute cortical necrosis was also reported in a forty-year old lady who was reportedly stung once on her hand by a wasp [126]. The beneficial potential of peptides obtained from wasp venom like mastoparan, melittin and apamin could lead to the development of anticancer and antimicrobial agents which would surely provide health benefits to man.

Ants venom consists of piperidine alkaloids and proteins which can cause potentially life-threatening allergic reactions in humans [127]. Clinicians reported that a fifty nine-year old man developed rhabdomyolysis resulting in acute renal failure due to envenomation from extensive fire ant bites, pointing to complications that could result from ant envenomation [128]. Anaphylaxis was reported in a child exposed to fire ant envenomation [129]. A twenty one-year old man developed acute renal failure caused by haemolytic-uremic syndrome due to fire ants bites [130]. A seventy-year old man bitten by a large number of ants presented with numerous pustules all over his body with itching and pain complains [131]. Solenopsins obtained from fire ant venoms could be beneficial to human health as it possesses insect repellant, insecticidal and necrotic properties in addition to other alkaloids present in fire ant venoms with reported antimicrobial activity against gram-positive bacteria [13]. Also, ant venoms contain a number of homo- and hetero- dimeric peptides with pore forming activities that could be utilized for the development of drug candidates [132].

Spider venoms stimulate a hyper-inflammatory response in humans through the release of pro-inflammatory cytokines that sometimes results in multiple organ dysfunction, fever, anorexia, hypotension and mortality [111]. Clinicians in Michigan, USA observed pruritus, redness, swelling, severe pain, fever, nausea and diarrhea in a fifty-nine year old woman who was bitten by a brown recluse spider, pointing to morbidities that could be caused by spider venom [133]. A nineteen-year old man bitten by brown recluse spider presented with fever, nausea, pain and dehydration to the extent that required intravenous fluid administration [134]. A retrospective study carried out on 59 patients showed elevated white blood cell count and impaired hepatic and renal function in addition to severe pain, inflammation and redness at the site bitten by black widow spiders [135].

Although mortality cases arising from centipede bites are rare, severe pain, swelling, lethargy, headache, dizziness and localized necrosis are most commonly reported among patients [136]. In relatively fewer cases, hemorrhage, tissue necrosis, nausea, vomiting, general rash, myocardial ischemia and infarction are reported [109]. Clinicians observed swollen lips, throat pain and shortness of breath in a twenty-one year old lady who suffered centipede envenomation [137]. An elderly man who suffered centipede envenomation presented with lymphangitis and dermatitis as complications associated with red headed centipede bite, while rhabdomyolysis and myocardial infarction were observed in other patients [138]. A twenty-year old man who was bitten by centipede presented with swelling, cold sweating, nausea, vomiting and chest pain with signs of acute myocardial infarction [139]. In another case report, a thirty one-year old man bitten by a centipede experienced swellings and pain with myocardial infarction and cardiopulmonary arrest observed later [140]. The potential for centipede venom use for medicinal purposes lies in the disulfide-rich proteins extracted from the venoms, which have antioxidant properties and could be utilized for drug discovery [119]. This is evidenced from reports which show that centipedes have been used for many decades in traditional Chinese and Korean medicine for the treatment of oxidative stress and inflammation-mediated disorders [104]. Also, considering the effect of neurotoxins from centipede venoms on ion channels, these compounds could be modified and used for the development of xenobiotics for beneficial purposes [110].

Scorpion venoms cause severe morbidity and mortality annually in humans due to the potent neurotoxins and bioactive peptides contained in the venoms [141]. A twenty four-year old woman who was stung by a scorpion experienced severe pain, tachycardia, tachypnea, hypotension, myocarditis, hypoxaemia and pulmonary edema which were managed until the patient was restored to health and discharged [142]. A nineteen-year old man stung by a scorpion experienced severe pain, nausea, profuse sweating, vomiting, abdominal pain, myocarditis and cardiogenic shock which were managed successfully [143]. Unfortunately, clinicians reported a fifty four-year old woman, bitten by scorpion and presented with progressive conditions of hypotension, myocarditis, transient ventricular tachycardia and sudden cardiac arrest which eventually led to her death [144]. A retrospective study on autopsy findings from scorpion sting-mediated mortalities showed that mechanism of death was mostly related to cardiotoxicity and terminal pulmonary edema due to the presence of neurotoxins, acidic proteins and various other organic compounds with known neurological and cardiovascular toxicities [145]. The most promising drug candidate obtained from scorpion venom is chlorotoxin, a 36-amino acid peptide, currently undergoing clinical trial for cancer management due to its ability to bind to tumor cells [141]. Peptides obtained from scorpion venom are also undergoing investigation for the development of antibacterial and antifungal agents which could be beneficial for human health [141].

Bee sting also affects animals with reports of erythemia, swellings, necrosis and skin damage especially on the ears observed in cattle stung by a swarm of bees [146]. Bee sting in a sheep caused mortality with pathological examination revealing dermal necrosis and hemorrhages in the heart and spleen [147]. A dog stung by a swarm of bees presented with hypotension, hypoglycemia, bradycardia, edema and erythema which were stabilized by the clinicians until the dog was restored to health and discharged [148]. A German shepherd dog that suffered multiple bee stings on different parts of the body presented with bloody vomiting and hematuria resulting in seizure and cardiac arrest, which eventually led to death [149]. The effect of bee stings on avian species was also reported with ten deaths within 24hours out of sixteen pigeons that suffered multiple bee stings [150]. Collectively, these reports show that bee stings affect the health of a range of animals. Among Hymenopterans, there are relatively fewer scientific reports on wasps and ant stings in animals, which necessitate future research attention due to the reported potency of their venoms in humans [106].

Spiders are generally considered as polyphagous with choice dependent on availability and susceptibility of target prey [151]. Spiders prey on aphids and arthropods [151]. Spiders also use pheromones to ensnare and consume moths [152]. Spiders utilize the silk they produce to trap diverse types of prey and use the captured prey for food [153]. Spiders that inhabit grasslands and forests are the major contributors for spider-associated prey kills [154]. Remarkably, lizards are resistant to spider venom and even prey on spiders [16]. Centipedes can kill using their venom and feed on vertebrates, such as bats, rats, amphibians and reptiles [109]. Crickets, houseflies and cockroaches are susceptible to centipede envenomation resulting in paralysis [19]. Scorpions envenomation in dogs is widely reported and is associated with elevated arterial pressure, heart rate and cardiac output with corresponding elevations in plasma epinephrine and norepinephrine levels [155]. Scorpion envenomation in dogs can cause significant changes to systemic blood pressure and cardiac output within 1 hour [156]. A retrospective study on scorpion envenomation in dogs reported lameness in one of the limbs, but mortality was not recorded [157].

### 5.2 Effect of insect toxins on the environment

The effects of insect toxins on the environment could be attributed to predator–prey relationships that could affect the delicate balance in the ecosystem [16]. Pigeons serve as food for hawks and falcons and play roles in seed dispersal. The susceptibility of pigeons to bee venom could have effects on food availability across this food chain [150]. Aphids are important herbivores and transmit economically important diseases while moths are important for pollination. Both aphids and moths are susceptible to spider envenomation with possible consequences on the ecosystem [158]. The delicate balance currently enjoyed in the ecosystem could be affected if climate change conditions favor the proliferation of certain predators as against certain preys. Considering that some insects are more present in certain territories than others, effective strategies are needed to protect humans, animals and the environment, so as to ensure the delicate balance in the ecosystem is sustained [159]. Also, venoms obtained from insects could be used to develop therapeutics that could be beneficial to humans, animals and the environment, thereby contributing to sustainable One Health [141].

## 6 Toxins from aquatic species and One Health

Toxins from aquatic species received extensive research interest when gastrointestinal morbidities were observed in people who consumed contaminated fresh water and sea foods [160]. Generally, these toxins are produced by planktons and algae that find their way into the food chain when continuously consumed and bio-accumulated in marine animals and subsequently in humans who consume these contaminated marine animals [161,162]. Herein, we focus on toxins produced by or present in aquatic cyanobacteria, algae, shellfish and toads.

Cyanotoxins are a class of toxins produced by cyanobacteria, which are prokaryotic photosynthetic organisms that are largely aquatic [163]. The most reported cyanotoxins include microcystins, saxitoxins, anatoxins and cylindrospermopsin [164]. Microcystins are cyclic nonribosomal peptides that are produced by cyanobacteria in fresh water and lakes [165]. They are potent hepatotoxins that interact with serine/threonine residues in proteins, cause oxidative stress and can act as carcinogens by inhibiting protein phosphatases [14]. Saxitoxins are a class of cyanotoxins produced by eukaryotic dinoflagellates in marine waters and five genera of cyanobacteria (prokaryotes) in fresh water [166]. Saxitoxins are potent inhibitors of voltage-gated sodium channels, thereby interrupting nerve transmissions, causing hypotension, paralysis that sometimes results in death [167]. Anatoxins are water-soluble alkaloids produced by freshwater species of cyanobacteria [168]. Anatoxin-a is a main anatoxin analog that mimics acetylcholine by binding irreversibly to acetylcholine receptors in nerves and neuromuscular junctions, inducing changes that could cause damage to the liver, kidney, lungs and heart [169]. Cylindrospermopsin is a zwitterionic, water soluble, cyclic guanidine alkaloid produced by fresh water cyanobacteria that is resistant to sunlight and high temperatures, making it easy to accumulate in different organisms [92]. Cylindrospermopsin can accumulate in the liver and cause oxidative stress leading to DNA and lipid oxidation and reduced glutathione synthesis [170]. Interestingly, studies are beginning to examine how some cyanotoxins could be modified for the development of therapeutic agents [163].

Phycotoxins are potent natural toxins synthesized by algae or sea weeds that can bio-accumulate in the food web, causing poisonings that could kill aquatic organisms including fishes [77, 78]. Phycotoxins are generally heat stable, which increases the likelihood of human exposure and toxicity on consumption of phycotoxin-contaminated fishes [9]. The classes of phycotoxins include domoic acid, ciguatoxin, brevetoxin, tetrodotoxin, okadaic acid, azaspiracid and palytoxin groups [172]. Aquatic algae are phototrophic eukaryotic organisms that can be found in bodies of fresh as well as salt water [173]. Seafood poisoning is often associated with the occurrence of algal ‘blooms’, which occur during rapid algal proliferation, sometimes resulting in visible discoloration of water [171]. Phycotoxins produced by algae pose a threat to the ecosystem and cause economic losses due to sea foods contamination [171]. Phycotoxin mediated toxicity is a world-wide problem [174]. In addition to contamination of sea foods, phycotoxin-mediated toxicity could also result from drinking of water contaminated by poisonous algae [175] and skin contact with water contaminated by poisonous algae [172]. The symptoms reportedly associated with phycotoxin consumption include diarrhea and paralysis that could sometimes lead to death due to inadequate antidotes for phycotoxin poisoning [173]. Further research is needed to develop more sensitive phycotoxin testing tools, identify management strategies to combat phycotoxin poisoning in the ecosystem and determine therapeutic potentials of phycotoxins [171].

Shellfish consume dinoflagellate organisms which contain toxins that can bio-accumulate in the fish and cause poisoning to birds and humans who consume such contaminated shellfish [176]. Shellfish poisoning in humans is difficult to avoid because the contaminated fish do not show unusual signs and most of the toxins are resistant to heat [172]. Based on clinical syndromes, shellfish poisoning can be classified into paralytic shellfish poisoning, diarrhoeic shellfish poisoning, amnesic shellfish poisoning and neurotoxic shellfish poisoning. Currently, a number of “emerging toxins”, like palytoxins, yessotoxins and pectenotoxins are attracting research attention [177].

Toads are aquatic animals that synthesize and secrete toxins from their glands which are useful for protection from predators [178]. Toad toxins consist of different steroids, cardiac glycosides and biogenic amines which exert various physiological effects [18]. The symptoms associated with toad toxin poisoning range from gastrointestinal to cardiac-related disorders [18]. Bufadienolides and bufotoxins exert their effects by inhibiting the sodium-potassium ATPase pump of cardiac myocytes and act on the central nervous system. A study reported that toad venom could induce cardiotoxicity by altering the activity of protein kinases that stimulate inflammation regulatory proteins [179]

### 6.1 Effects of toxins from aquatic species on humans and animals

Cyanotoxin exposure has caused a number of reported toxicities which include gastroenteritis, hepatomegaly and cardiac disorders that sometimes resulted in fatalities [180]. Other reported effects of cyanotoxins in humans include paralysis, carcinogenesis and triggering of neurodegenerative diseases [181]. Microcystins-related poisoning in humans resulting in adverse health effects has been reported in Australia, Brazil and the United States [182]. Shellfish poisoning in humans and birds due to consumption of saxitoxin-contaminated shellfish results in neurotoxic effects, paralysis and sometimes death [160]. Exposure to anatoxins in humans and animals causes neurologic symptoms causing damage to muscles, heart and lungs [169]. It has been shown that animals are more affected in number with respect to cyanotoxins poisoning [183]. A review on *in vitro* toxicological assessment of cyclindrospermopsin showed that exposure to this toxin in humans and aquatic animals caused liver damage and death via mechanisms involving altered protein synthesis and apoptosis [184]. An *in vitro* study carried out in adult human stem cells showed that exposure to cyclindrospermopsin caused damage to hepatocytes via non-genotoxic means, but altered the activity of proteins required for cell survival [170]. A sub-chronic toxicity study carried out in Sprague–Dawley rats showed that exposure to cyclindrospermopsin caused damage to liver and kidney pointing to the potential toxicities that could result from long-term exposures at very low doses [185]

Contamination of seafoods with phycotoxins and the resultant effects on human health is a worldwide problem [186]. Human and farm animal intoxications caused by phycotoxins associated with gastrointestinal and hepatic disorders with reported cases of mortality [176]. Aquatic animals are significantly affected by phycotoxin poisoning [171]. Phycotoxin poisoning in humans and animals is associated with acute toxicity resulting in adverse health effects [162]. Economic losses due to contaminated sea foods can affect the economy of a nation, thereby constraining financial resources that could have been dedicated to providing health care facilities [171]. A study carried out using human cell model showed that phycotoxins could act synergistically with other toxins to cause gastrointestinal and neuronal toxicity in humans [187]. Further studies are needed in this regard especially with respect to human and animal exposure to multiple toxins at relatively low doses in regions prone to algae contaminated water.

Shellfish poisoning in humans is reported in many countries with significant morbidity and mortality [176]. In 2013, during the red tide, 58 cases of shellfish poisoning resulting in 4 deaths was reported among people who consumed shellfish bought from the market or picked up at the beach [188]. The most common symptoms observed in patients included dizziness, numbness, nausea and breathing difficulties [188]. In Alaska, dysphagia and dysarthria were observed in patients who suffered from shellfish poisoning [189]. In the Philippines, 31 cases of shellfish poisoning and 2 deaths were recorded among locals who consumed green mussels with sea water samples showing presence of saxitoxin [190].

Toad poisoning in humans resulting in mortality was reported in Thailand with patients who consumed toad meat showing gastrointestinal disorder, bradycardia and cardiac arrest [18]. Toad consumption for medicinal purpose is practiced in parts of China and India, with reports of bradycardia, cardiac dysfunction and death in some cases [191]. A patient exposed to toad poison experienced burning sensation in the eyes, ocular hypotonia and loss of vision [192]. Toad poisoning in birds causes necrosis in liver and heart and hemorrhage in the lungs and brain [178]. A fur seal in a German zoo was found dead in its outdoor enclosure with further autopsy examination revealing an ingestion of two toads while toxicological analysis of the stomach content showed presence of bufadienolides [193]. Toad poison in dogs, cats and toad-eating aquatic animals have also been reported [194]. Interestingly, alkaloids, steroids and peptides obtained from toad poison could have potential for development of therapeutics [195].

### 6.2 Effects of toxins from aquatic species on the environment

**Fig. 2:**
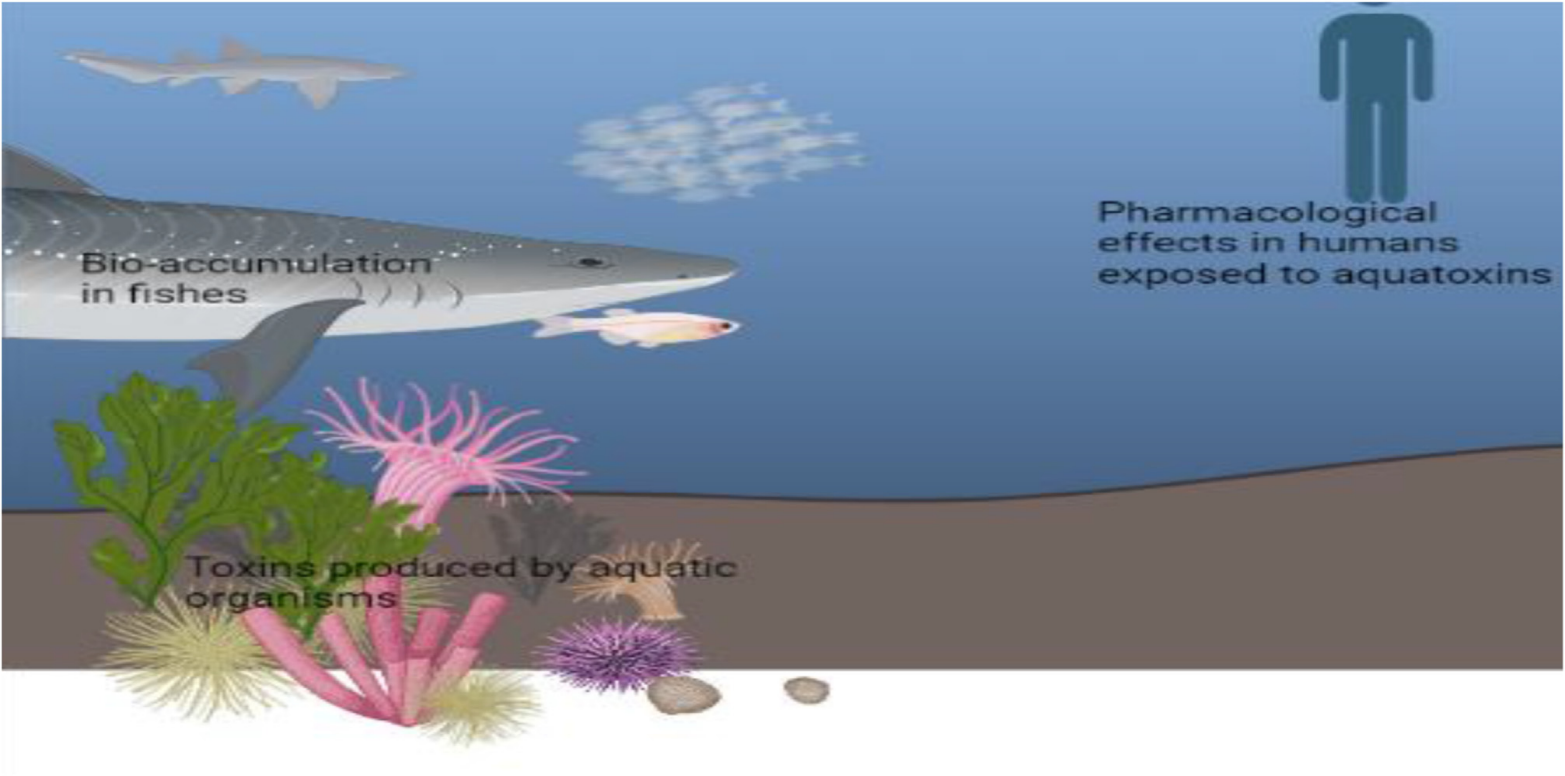
Aquatic toxins in humans, animals and the environment.

A number of cyanotoxins produced by cyanobacteria are water-soluble. They can contaminate water bodies and soil, get absorbed by aquatic plants with a potential for bio-accumulation and be transferred to higher organisms in the ecosystem as illustrated in Fig. 2 [164]. The same scenario is possible when plants are irrigated with water from cyanobacteria-contaminated streams, fresh water rivers and marine water. A study on microcystins shows that this toxin affects soil bacteria function, impaires soil nitrification and causes toxicity to tomato, wheat and lettuce plants [196]. A similar study reported that exposure to microcystins retarded growth performances of garden peas, wheat and maize in a dose- dependent manner [165]. An *in vivo* study carried out on earthworms showed that exposure to microcystins induced oxidative stress and affected metabolism, which could have effects on ecosystem balance due to the role of earthworms in soil ecology [197]. Anatoxins contaminate water bodies and can be absorbed by aquatic plants as evidenced by a study which showed that oxidative stress and impaired growth was observed in aquatic plants exposed to anatoxin-a [198].

Phycotoxins can also threaten ecosystem stability through deleterious effects observed in many aquatic organisms [199]. Growth conditions that promote proliferation of algae species could increase phycotoxin levels in water bodies and contribute to related toxicity [199]. Fresh, brackish and seawater contaminated with phycotoxins cause adverse effects on zooplanktons and reduce their population which could affect the ecosystem [200]. Phycotoxins produced by algae could contribute to reduced water quality by increasing turbidity and affecting dissolved oxygen levels [199].

Studies are required to examine if toad toxins are water soluble, affect the growth of aquatic flora and soil organism which are important contributors to ecosystem balance. Global machineries that holistically address management tools and regulatory policies for monitoring and controlling the effects of aquatic toxins on soil, water, animal and human health are required. Public awareness strategies that take into account of local sources of aquatic toxins and cultural practices that put certain toxin-producing aquatic organism at risk need to be put in place to protect human, animal and aquatic organism populations. Clearly, the cooperation of different classes of professionals in this regard is required.

## 7 Reducing the menace of natural toxins to ensure One Health

The measures and strategies for reducing the detrimental effects of natural toxins have focused more on humans and animals; identifying sources, evaluating effects, mechanisms of action and adopting measures to reduce the severity associated with exposure [171]. However, improvements are needed in terms of robust regulation and policy formulation, improved surveillance of water bodies and lifestyle changes where necessary to reduce exposure to toxins [201]. Deliberate strategies aimed at ecosystem preservation such as breeding of crop varieties with lower phytotoxin content could be beneficial [5]. Where necessary, phytoremediation can also be used to reduce the effects of identified phytotoxins [202]. Developing countries need to improve human and animal health care facilities. Improvements are also needed on creating awareness among the populace on the dangers as well as benefits of natural toxins. Health professionals need to rise to the challenge through collaborative and synergistic efforts towards building a defensible and healthy society [203].

Also, a One Health approach should be increasingly applied in decision-making. While the application of One Health approaches relies on strong political commitment, establishment of governmental agencies specifically responsible for the coordination of a One Health approach is encouraged. It has been observed that inadequate institutional structures for capacity building constitute a challenge to the One Health approach. Thus, there is a need for more conducive political environments that stimulate and promote multi-sectoral and transdisciplinary cooperation at all levels [31]. These measures could lead to a more resilient environment that contributes positively to the health of humans and animals. Potential detrimental effects of toxins to achieve One Health is depicted in Table 1.

**Table 1:** Potential detrimental effects of toxins to achieve One Health.

Toxin	Effect on humans, animals and the environment	Strategies to address observed effects	Research gaps	References
Phytotoxins	- Phytotoxins induce liver damage, including hepato-sinusoidal obstruction syndrome and veno-occlusive liver disease - Causes reproductive dysfunction (abortion and fertility status). - It is also reported to have carcinogenic properties e.g. safrole. - They also contribute to soil-borne diseases that inhibit the growth of commercial crops and economic plants in the ecosystem.	- Adoption of interrelated behavioral and physiological strategies to reduce the risk of poisoning such as: detoxification, changes in diet selection etc - Phytoremediation (to reduce soil contamination via toxins), trans location, and detoxification of plants. - Preservation of nature, restoration of ecosystem, breeding of crop varieties with lower phytotoxin content and a more extensive agricultural practice.	The wide varieties of phytotoxins and its effect on animal, human and the environment is attracting attention from scholars and researchers. Thus, providing an opportunity to further research on deep understanding of these phytotoxic compounds and their unknown effects on humans, animals and the environment.	[5,5,32,36,37,44,58,59,65,98,202,204,205]
Microbial toxins	- Microbial toxins interfere with neurotransmission of the cholinergic type leading to eventual death by failure of peripheral mechanisms (respiratory failure and cardiac arrest) - Some studies have implicated the role of toxins in carcinogenesis. - In the environment, they cause decreased water quality due to the production of off-flavors, discoloration of water, and accumulation of surface scums.	improvements in antimicrobial use, better regulation and policy, as well as improved surveillance, stewardship, infection control, sanitation, and finding alternatives and lifestyle change	it is obvious however, that while the animal and human aspects are significantly given optimum attention, the impact of toxins on the environment is not as well studied in comparison.	[9,88,91,201]
Mycotoxins	-Primarily affect liver and induce damage in hepatocytes and tissues mainly through cell cycle arrest and inhibition of cell proliferation, and the induction of apoptosis, oxidative stress, endoplasmic reticulum stress and autophagy (e.g. aflatoxins) - Mycotoxin such as ochratoxin has been shown to be nephrotoxic, hepatotoxic, teratogenic and immune-toxic. - Kidney and liver tumors - Air pollution.	Effective sanitary measures, improved human health care facilities, awareness creation, improved prevention and control efforts.	Many countries are well aware of mycotoxin and have developed regulations (through Food regulatory bodies) governing the acceptable concentration in human and animal feed. However, its acceptable concentration in the environment is unexplored.	[96,98,203].
Phycotoxins	- Acute illness and serious health risks in humans; Respiratory, gastrointestinal, neurological, dermatological, and musculo-skeletal symptoms. - Phycotoxins contaminate fresh, brackish and seawater. - Reduction of population quantity. - Non-toxic HABs lead to the water quality loss by an excessive increase of turbidity and dissolved oxygen consumption.	- The key management tools are prevention through policies, regulations, routine monitoring of water bodies and public awareness. - Collaborative and synergistic efforts towards building a defensible and healthy society by the health professionals. - Interdisciplinary interaction and collaboration among all health professionals.	There is no established systematic approach to removing most of the toxins in our environment.	[97,171,176,186,203,206].

## 8 Conclusion

The One Health concept was necessitated by the increasing global threat from diseases that highlight a unique interface of humans, animals and the environment, as well as the need for an integrated approach to managing this. Due to the exposure of humans and animals to a wide range of natural toxins in the environment, there is growing interest about the potential effects such interactions could have on the health of humans and animals. Some natural toxins pose risks to human and animal health through contamination of the environment. Nevertheless, some have beneficial effects and could be used for the development of novel therapeutics. The connection between humans, animals and the environment necessitates the collaboration of different professionals to comprehensively understand and reduce risks and consequences of toxins on the environment, animals and humans. Adopting holistic mechanisms that counteract the detrimental effects of natural toxins and optimize their benefits will improve global health. Clearly, a One Health approach to natural toxin management provides a powerful platform to create a healthier and sustainable globe for everyone.

## Author contributions

Conceptualization: GE Forcados; Writing-original draft preparation: AR Nwaji, O Arieri, AS Anyang, K Nguedia and EB Abiade; Supervisors: GE Forcados, OO Oladipo, S Makama, IL Elisha, N Ozele and JG Gotep; Funding acquisition: OO Oladipo; Writing-reviewing and editing: IL Elisha, GE Forcados, AR Nwaji and O Arieri. All authors have read and agreed to publish this version of the manuscript.

## Conflict of interest

The authors declare no conflict of interest
